# Genomic characterization of norovirus and Sapovirus from a diarrhea outbreak in a school linked to heavy rainfall

**DOI:** 10.3389/fmicb.2025.1570161

**Published:** 2025-04-30

**Authors:** Zhongyan Fu, Changjing Wu, Yinghui Yu, Jian Zhao, Yan Dong, Wei Liu, Huixin Dou, Xuezhen Shi, Chengzhi Cai, Baihai Jiao, Tiantian Liu, Boyan Jiao

**Affiliations:** ^1^Department of Infectious Disease Control, Shandong Center for Disease Control and Prevention, Jinan, China; ^2^Shandong Provincial Key Laboratory of Intelligent Monitoring, Early Warning, Prevention and Control for Infectious Diseases, Jinan, China; ^3^Department of Laboratory, Jining Center for Disease Control and Prevention, Jining, China; ^4^Jining Key Laboratory of Infectious Disease Control and Prevention, Jining, China; ^5^Department of Infectious Disease Control, Jining Center for Disease Control and Prevention, Jining, China; ^6^Department of Infectious Disease Control, Sishui Center for Disease Control and Prevention, Sishui, China; ^7^Gynecology Department, Baoding First Central Hospital, Baoding, China; ^8^Computer Science Department, Central Connecticut State University, New Britain, CT, United States; ^9^Department of Medicine, School of Medicine, University of Connecticut Health Center, Farmington, CT, United States; ^10^Department of School Health, Jining Center for Disease Control and Prevention, Jining, China

**Keywords:** heavy rainfall, diarrhea outbreak, Norovirus, Sapovirus, whole genome

## Abstract

**Background:**

Diarrhea poses a serious threat to human health, and rainfall is known to increase the incidence of diarrheal diseases. On July 7, 2024, a diarrhea outbreak occurred in a school in Sishui County, Jining City, Shandong Province, China, following heavy rainfall. This study aimed to identify the pathogens responsible for the outbreak and characterize their whole genomes.

**Methods:**

On July 8, 2024, a total of 21 stool samples from diarrhea cases, 2 water samples from private wells, and 1 drinking water sample from the school cafeteria were collected. Real-time quantitative PCR was used to detect Rotavirus A (RV-A), Norovirus genogroup I (NV GI), Norovirus genogroup II (NV GII), Sapovirus (SaV), Human Astrovirus (HAstV), and Human Adenovirus (HAdV). Whole-genome sequencing was performed for NV GI and SaV-positive samples to determine their genotypes, construct phylogenetic trees, and analyze amino acid variation sites in encoded proteins.

**Results:**

Among the 21 case stool samples, 7 tested positive for both NV GI and SaV, 10 were positive for NV GI only, and 1 was positive for SaV only. Of the 2 private well water samples, one was positive for NV GI and the other for SaV. Whole-genome sequences were obtained for 11 NV GI strains and 2 SaV strains. The 11 NV GI sequences from the outbreak exhibited high homology, with whole-genome similarity ranging from 99.96% to 100%, and were all identified as the NV GI.6 [P11] genotype. Phylogenetic analysis showed that these 11 sequences clustered within the same evolutionary branch. Similarly, the 2 SaV sequences were highly homologous, with 99.97% similarity, and were identified as the SaV GI.6 genotype, clustering within the same phylogenetic branch.

**Conclusions:**

This diarrhea outbreak was caused by the combined presence of NV GI and SaV following heavy rainfall. These findings provide valuable reference data for the prevention and control of diarrhea outbreaks caused by heavy rainfall or multiple pathogens.

## 1 Introduction

Diarrhea is a significant global public health issue, causing approximately 1.17 million deaths annually worldwide and ranking as one of the leading causes of child mortality (GBD 2021 Diarrhoeal Diseases Collaborators, 2024). The causes of diarrhea are diverse, with viral infections, such as Norovirus (NV) and Sapovirus (SaV), being major contributors (Cohen et al., 2022). NV accounts for an estimated 680 million diarrhea cases each year and approximately 200,000 deaths annually (Winder et al., 2022). SaV is responsible for about 10% of diarrhea cases and is estimated to cause 23,000 deaths annually among children under the age of five (Cohen et al., 2022; Pang et al., 2000).

Both NV and SaV belong to the Caliciviridae family. The NV genome contains approximately 7,500 nucleotides (Lin et al., 2023), comprising three open reading frames (ORFs). ORF1 encodes a polyprotein that is cleaved into six non-structural proteins, including p48, NTPase, p22, VPg, Pro, and RdRp. ORF2 encodes the structural protein VP1, while ORF3 encodes the structural protein VP2 (Lin et al., 2023). Based on the sequences of the VP1 and RdRp genes, NV is classified into G (genogroup) and P (polymerase) genotypes (Chhabra et al., 2019), with GI and GII being the primary genogroups infecting humans (Winder et al., 2022; Chigor et al., 2024; Wu et al., 2024).

The SaV genome consists of 7,100–7,700 nucleotides and generally contains two open reading frames (ORFs). ORF1 encodes a polyprotein that is cleaved into six non-structural proteins (NS1, NS2, NS3, NS4, NS5, and NS6-7) and the structural protein VP1, while ORF2 encodes the structural protein VP2 (Oka et al., 2015; Yinda et al., 2017). Based on the VP1 coding region sequence, SaV can be classified into different genotypes (Oka et al., 2012), with GI, GII, GIV, and GV being the primary genotypes infecting humans (Zhao et al., 2024).

The occurrence of diarrhea is closely related to environmental and climatic factors, as heavy rainfall can lead to the contamination of water sources with pathogens like NV (de Man et al., 2014; Joosten et al., 2017), thereby increasing the incidence of diarrheal diseases (Levy et al., 2016; Dhimal et al., 2022; Azage et al., 2017). On July 7, 2024, a heavy rainfall event occurred in Sishui County, China, with precipitation exceeding 240 mm. Between July 7 and July 9, a diarrhea outbreak involving 26 cases was reported in a local school in Sishui County. In this study, stool samples from diarrhea cases and water samples were tested for RV-A, NV GI, NV GII, SaV, HAstV, and HAdV. The results identified NV GI and SaV as the causative agents. Whole-genome sequencing and analysis of NV GI and SaV were performed, providing insights to inform the monitoring, prevention, and control of diarrheal diseases following heavy rainfall.

## 2 Materials and methods

### 2.1 Sample collection

From July 7 to July 9, 2024, a diarrhea outbreak characterized by nausea and vomiting as the primary clinical symptoms was reported at a school in Sishui County, Jining City, Shandong Province, China. A total of 26 cases were identified, with 12 cases on July 7, 9 cases on July 8, and 5 cases on July 9.

On July 8, 2024, 21 stool samples (each weighing more than 5 g) were collected from affected individuals. Additionally, 2 private well water samples and 1 cafeteria drinking water sample (each 500 mL) were collected. All samples were sent to the Microbiology Laboratory at the Jining Center for Disease Control and Prevention for testing.

### 2.2 Nucleic acid detection

The nucleic acid detection protocol was based on our previously described method (Wu et al., 2024). Briefly, 0.1 g of stool sample was mixed with 0.9 mL PBS and vortexed three times. The mixture was centrifuged at 8,000 g for 5 min. After filtering 500 mL of water using medium-speed qualitative filter paper, MgCl_2_ was added to achieve a final concentration of 0.05 mol/L, adjusting the pH to 3.0–3.5. The suspension was filtered through a 0.45 μm nitrocellulose membrane using a large-volume pathogen concentrator (Beijing ZC Bio, Model: PurLVS), and the membrane was shaken in 1 mL PBS, and after vortexing and allowing it to stand, the supernatant was extracted. A 200 μL aliquot of the supernatant was used for nucleic acid extraction with a fully automated nucleic acid extraction instrument (GeneRotex 96, Xi'an Tianlong Technology Co., Ltd.) and a viral nucleic acid extraction kit (Cat. No. T138, Xi'an Tianlong Technology Co., Ltd.).

Real-time quantitative PCR was performed using triplex nucleic acid detection kits for RV-A, NV GI, and NV GII (Cat. No. A2593YH, Beijing ZC BioScience Co., Ltd.) and for SaV, HAstV, and HAdV (Cat. No. A2723YH, Beijing ZC BioScience Co., Ltd.).

### 2.3 Genome sequencing

Nucleic acid samples positive for NV GI and SaV were sent to Shanghai BioGerm Technology Co., Ltd. for whole-genome sequencing. NV-specific target gene amplification was conducted using the NV whole-genome targeted capture kit (Shanghai BioGerm Technology Co., Ltd.), followed by library construction using a whole-genome DNA library construction kit (Shanghai BioGerm Technology Co., Ltd.). Sequencing was performed using the DNBSEQ-T7 PE150 platform (Shenzhen BGI Technology Co., Ltd.).

### 2.4 Genetic sequence analysis

Genotyping of NV GI sequences was conducted using the Norovirus Typing Tool Version 2.0 (https://www.rivm.nl/mpf/typingtool/norovirus/), while SaV sequences were genotyped using the Human Calicivirus Typing Tool (https://calicivirustypingtool.cdc.gov/). BLAST analyses were performed against the NCBI database. Full-genome sequences of NV GI.1 [P1], GI.2 [P2], GI.3 [P3], GI.3 [P13], GI.4 [P4], GI.5 [P5], GI.5 [P4], GI.6 [P6], GI.6 [P11], and GI.7 [P7] genotypes and SaV GI.1, GI.2, GI.3, GI.5, GI.6, and GI.7 genotypes were downloaded from the NCBI database. Sequence alignment was conducted using MEGA 7.0.14, and phylogenetic trees for the NV whole genome, ORF1, ORF2, ORF3, and SaV whole genome, ORF1, and ORF2 were constructed using the maximum-likelihood method with 1,000 bootstrap replicates. Phosphorylation sites in proteins were analyzed using NetPhos-3.1 (https://services.healthtech.dtu.dk/services/NetPhos-3.1/), and N-linked glycosylation sites were analyzed using NetNGlyc-1.0 (https://services.healthtech.dtu.dk/services/NetNGlyc-1.0/).

## 3 Results

### 3.1 Epidemiological characteristics and nucleic acid testing results

**Figure 1:**
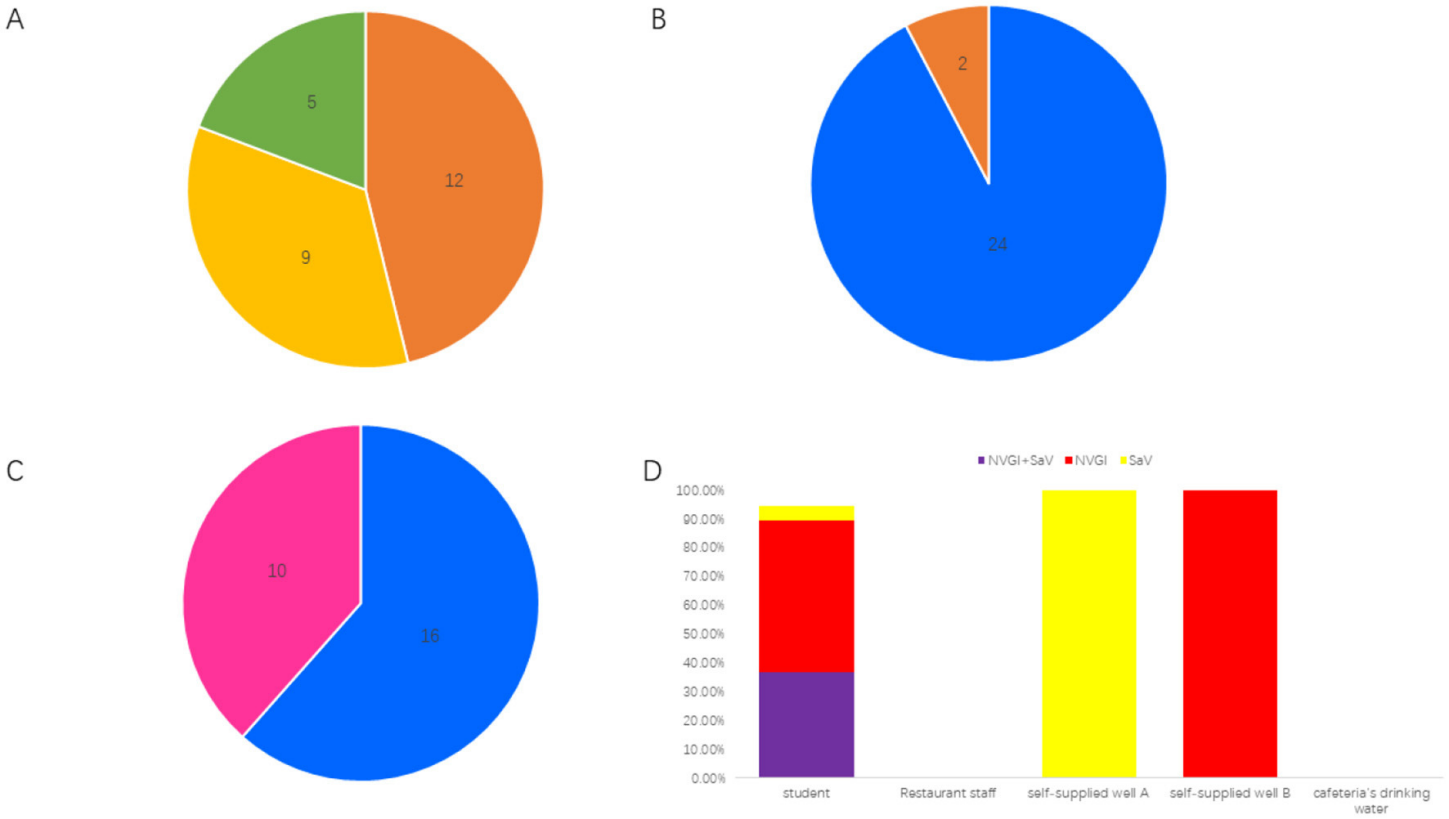
Epidemiological characteristics of the diarrheal outbreak. **(A)** Distribution of onset times; a total of 12 cases were reported on July 7, 2024, 9 cases on July 8, 2024, and 5 cases on July 9, 2024. **(B)** Distribution of affected populations; there were 24 students and 2 restaurant staff members. **(C)** Gender distribution; there were 16 male cases and 10 female cases. **(D)** Pathogen detection results indicated that among 19 student cases, the positivity rate for both NVGI and SaV viruses was 36.84% (7/19), the positivity rate for NVGI only was 52.63% (10/19), and the positivity rate for SaV only was 5.26% (1/19). Both samples from restaurant staff and the cafeteria's drinking water tested negative. However, self-supplied well A tested positive for SaV, and self-supplied well B tested positive for NVGI.

The school affected by the infectious diarrhea outbreak includes 26 classes across the 1^st^ and 2^nd^ years of senior high school, with approximately 1,500 students and 120 staff members. A total of 26 cases of diarrhea were reported during this outbreak, all of which presented symptoms of nausea and vomiting, with some also experiencing diarrhea and fever. The first case was reported at 16:00 on July 7, 2024, with a total of 12 cases occurring on that day, 9 cases on July 8, and 5 cases on July 9. Among the patients, 24 were students aged 16 to 18, distributed across 9 classes; 2 cafeteria staff members were aged 39 and 41, respectively. The gender distribution was 16 male cases and 10 female cases (Figures 1A–C).

The school has two self-supplied wells: Well A provides water for the public restrooms in the school office building, while Well B supplies water for the student dormitories and public restrooms in the teaching building. The daily water supply for the school cafeteria and drinking water for all students and staff is sourced from the city water supply of Shou County. On July 7, 2024, Shou County experienced heavy rainfall exceeding 240 mm, which led to the backflow or leakage of sewage in both self-supplied wells.

A total of 21 fecal samples were collected from the cases involved, including 19 samples from students and 2 samples from cafeteria staff. Additionally, one sample each was collected from Well A, Well B, and the cafeteria drinking water for testing of NV, rotavirus, SaV, astrovirus, and adenovirus nucleic acids. Among the 19 student fecal samples, 7 tested positive for both NV GI and SaV, 10 tested positive only for NV GI, 1 tested positive only for SaV, and 1 tested negative. Both of the cafeteria staff fecal samples were negative. SaV was detected positive in Well A, NV GI was detected positive in Well B, while the cafeteria drinking water tested negative (Figure 1D).

### 3.2 NV whole genome sequencing analysis

#### 3.2.1 Similarity analysis

In this diarrheal outbreak, 11 complete NV GI genome sequences were obtained from the fecal samples of patients, with sequence lengths ranging from 7,682 bp to 7,702 bp. Unfortunately, Whole-genome sequencing of norovirus was conducted on the self-sourced well water, but only a 75-nucleotide fragment (positions 3,287–3,361) of the ORF1 region was obtained. The nucleotide sequence of this fragment from the self-sourced water was identical to the corresponding sequence from 11 NV GI strains involved in the diarrhea outbreak. A BLAST analysis of this 3,287**–**3,361 nucleotide sequence from the self-sourced water in the NCBI database did not reveal any exact matches. The closest match was with 18 norovirus GI.6 [P11] strains, showing a similarity of 98.67%, with a single nucleotide difference. Among the 11 NV GI sequences, 6 were found to be completely identical, and the sequences exhibited 7 nucleotide variations, with similarities ranging from 99.96% to 100%. The only differences in amino acid sequences among the 11 NV GI whole genome sequences were observed in the RdRp and VP1 regions, with amino acid similarities of 99.80% to 100% and 99.81% to 100%, respectively, while the amino acid similarities for P48, NTPase, p22, VPg, Pro, and VP2 were 100%, indicating complete identity.

BLAST analysis in the NCBI database revealed that the NV GI sequences from this diarrheal outbreak showed the highest similarity, ranging from 99.00% to 99.02%, to the Norovirus GI isolate 2019 (GenBank: MW243609.1) derived from human feces in Shanxi, China. Specifically, the ORF1 similarity was 98.92% to 98.96%, ORF2 similarity was 99.07% to 99.14%, and ORF3 similarity was 99.52%. Compared to MW243609.1, the amino acid sequences of Pro and VP2 exhibited 100% similarity, while differences were observed in the amino acid sequences of P48, NTPase, p22, VPg, RdRp, and VP1 (Table 1).

**Table 1:** Similarity analysis of NV GI genes.

**Gene**		**Similarity among the 11 NV GI sequences from the current diarrhea outbreak**		**Similarity of the 11 NV sequences from the current diarrhea outbreak with the 2019 isolate MW243609.1 from Shanxi, China**	
**Gene name**	**Protein name**	**Nucleotide**	**Amino acid**	**Nucleotide**	**Nucleotide**
Complete genome sequence		99.96%−100%	-	99.00%−99.02%	-
ORF1	p48	99.94%−100%	100%	98.92%−98.96%	99.75%
	NTPase		100%		99.72%
	p22		100%		99.50%
	VPg		100%		99.27%
	Pro		100%		100%
	RdRp		99.80%−100%		99.80%−100%
ORF2	VP1	99.94%−100%	99.81%−100%	99.07%−99.14%	99.81%−99.63%
ORF3	VP2	100%	100%	99.52%	100%

#### 3.2.2 Gene evolution analysis

**Figure 2:**
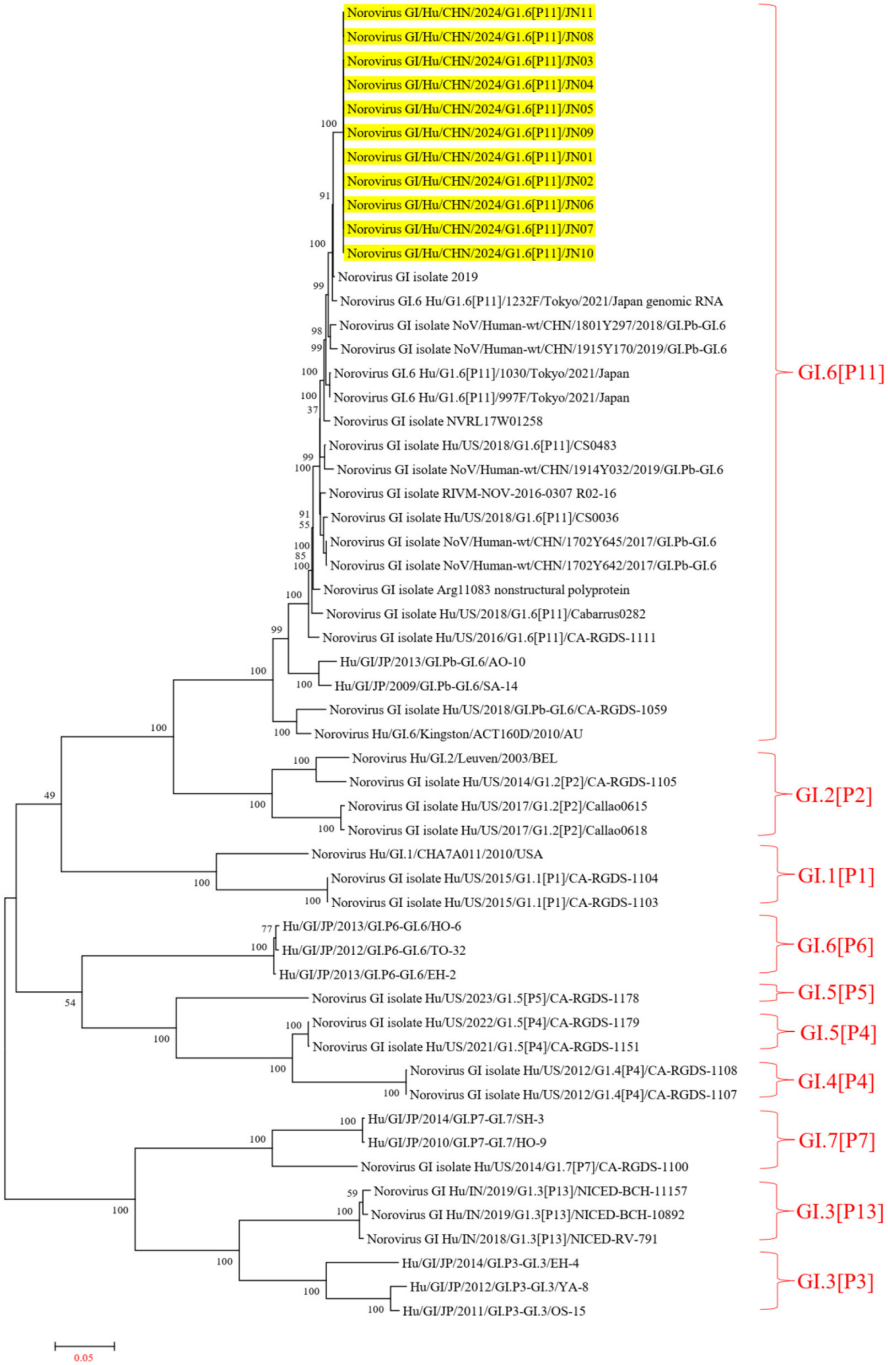
Phylogenetic tree of NV GI full genes. The phylogenetic tree was constructed using the maximum likelihood method with 1,000 bootstrap replicates, employing MEGA version 7.0.14 software. The yellow lines represent the NV GI sequences associated with this diarrheal outbreak.

The Norovirus Typing Tool Version 2.0 was utilized to analyze the genotypes of the 11 NV GI sequences from this diarrheal outbreak, and all 11 NV GI sequences were confirmed to be of the GI.6 [P11] genotype. Reference sequences for various genotypes, including GI.6P (Oka et al., 2012), GI.1P (GBD 2021 Diarrhoeal Diseases Collaborators, 2024), GI.2P (Cohen et al., 2022), GI.3P (Winder et al., 2022), GI.3P (de Man et al., 2014), GI.4P (Pang et al., 2000), GI.5P (Pang et al., 2000), GI.5P (Chhabra et al., 2019), GI.6P (Chhabra et al., 2019), and GI.7P (Chigor et al., 2024), were obtained from the GenBank database to construct an evolutionary tree. In the NV GI whole genome phylogenetic tree, the 11 NV GI sequences from this diarrheal outbreak formed an evolutionary cluster positioned within the GI.6 [P11] evolutionary branch (Figure 2).

**Figure 3:**
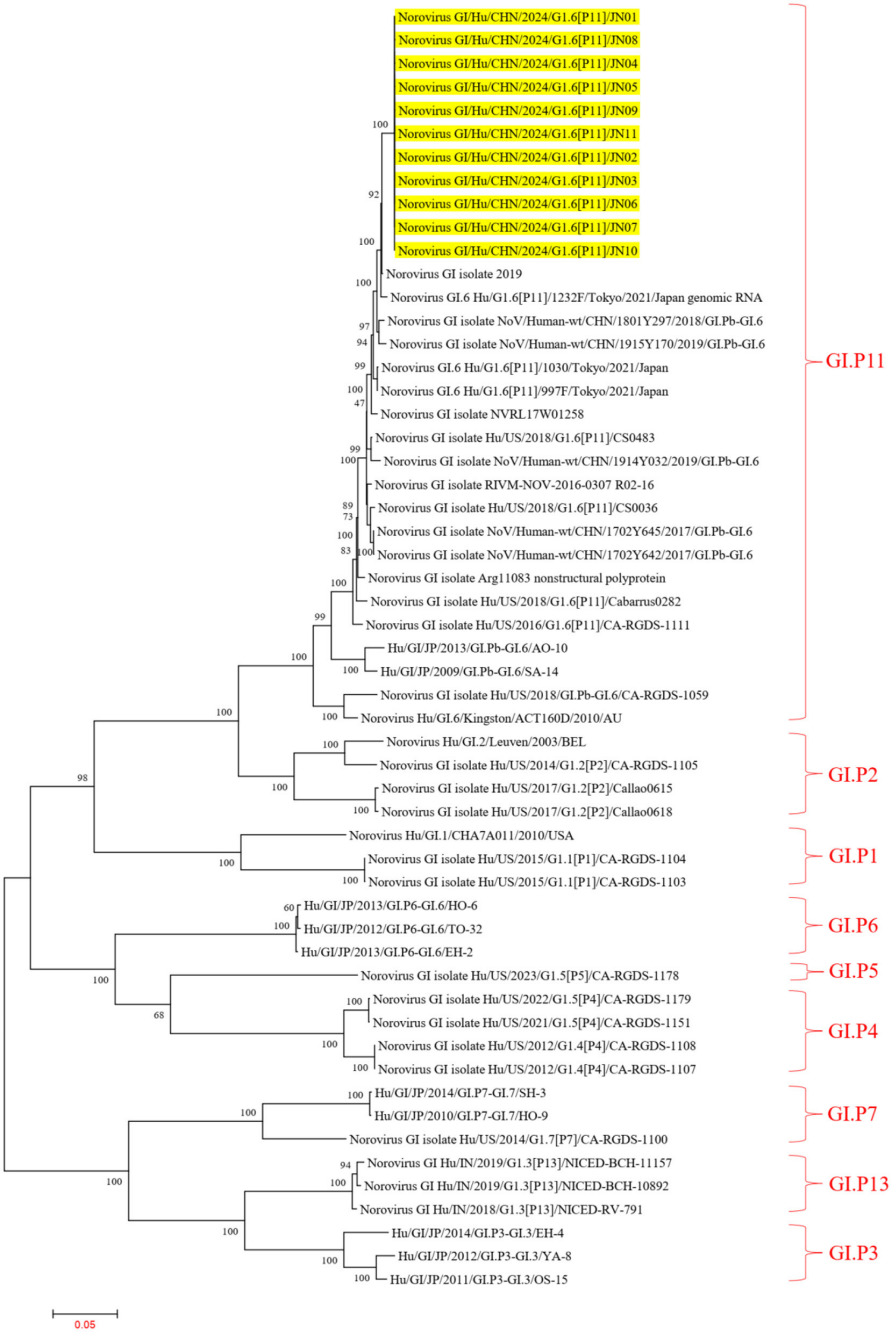
Phylogenetic tree of the ORF1 region of NV GI. The tree was constructed using the maximum likelihood method with 1,000 bootstrap replicates in MEGA 7.0.14 software. The yellow branches represent the NV GI sequences associated with the current diarrheal outbreak.

**Figure 4:**
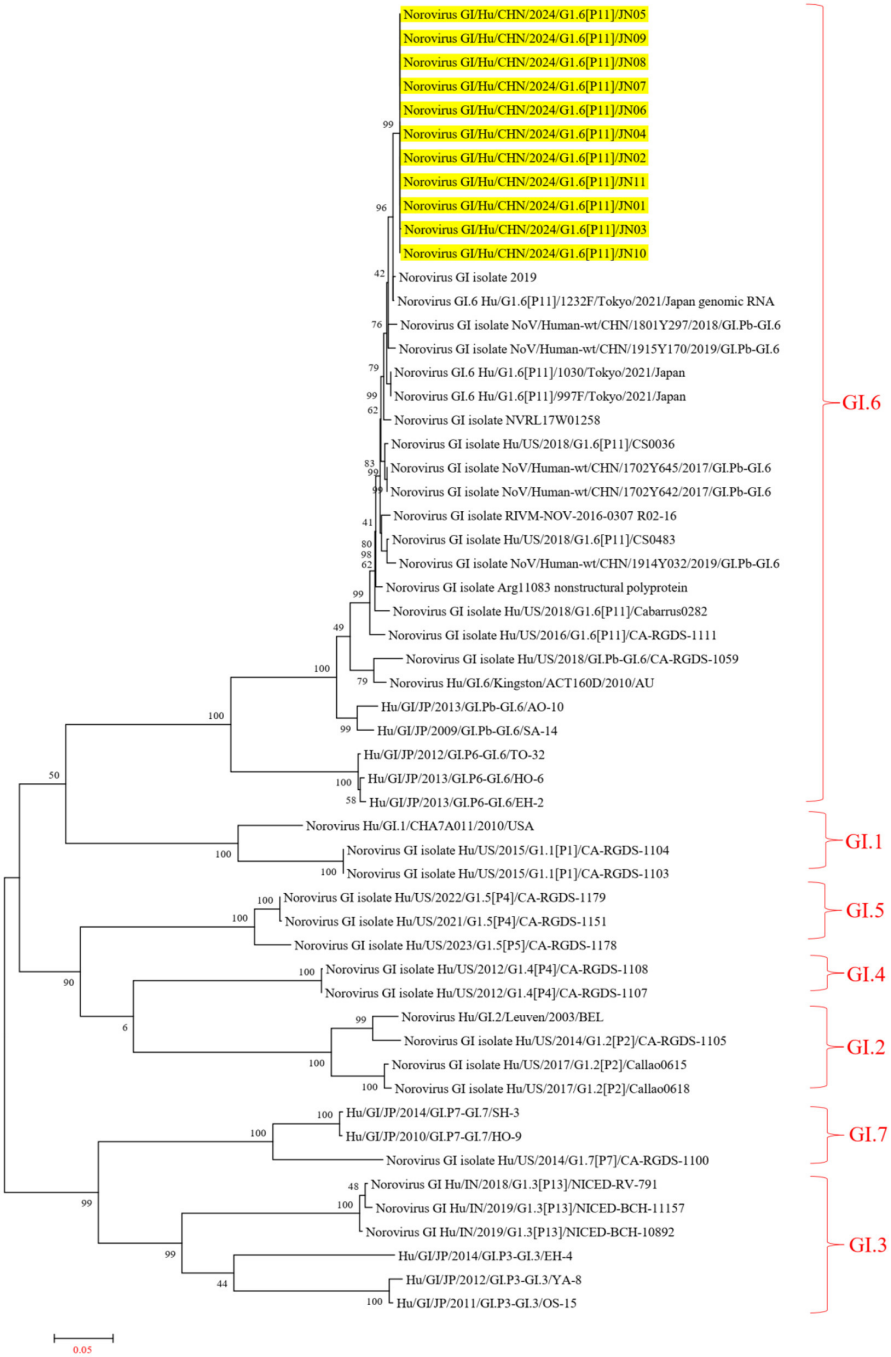
Phylogenetic tree of the ORF2 region of NV GI. The tree was constructed using the maximum likelihood method with 1,000 bootstrap replicates in MEGA 7.0.14 software. The yellow branches represent the NV GI sequences associated with the current diarrheal outbreak.

**Figure 5:**
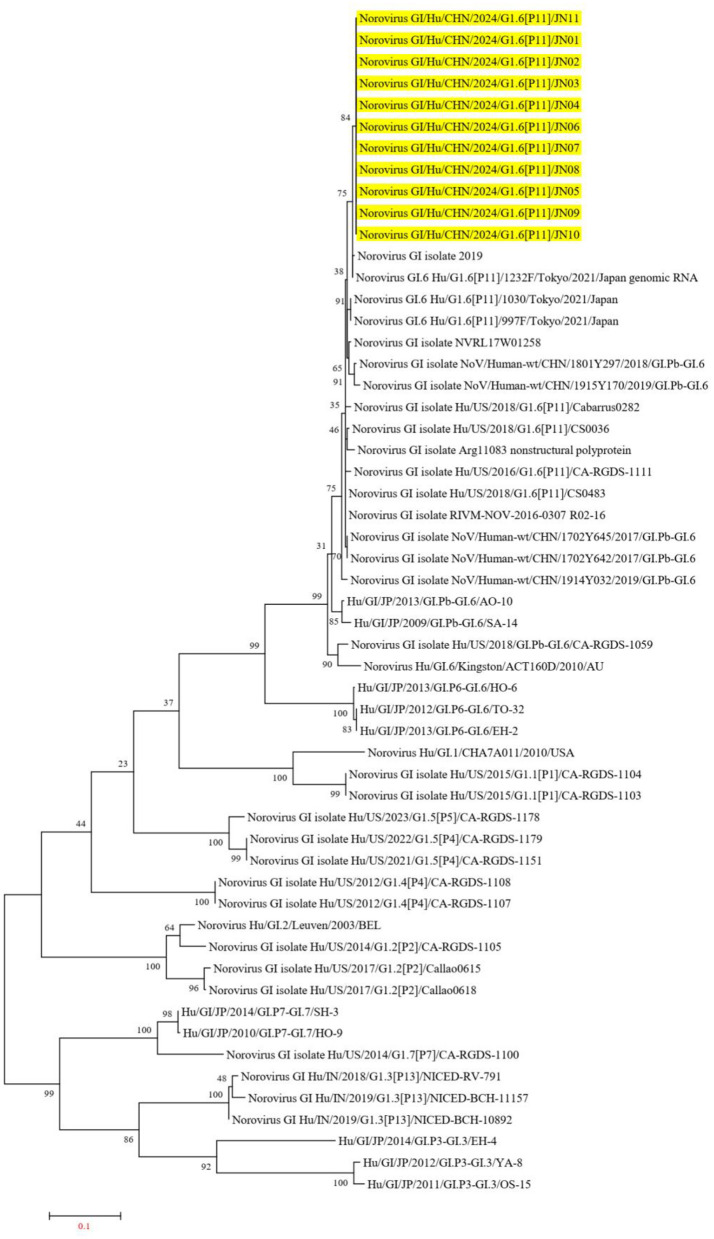
Phylogenetic tree of the ORF3 region of NV GI. The tree was constructed using the maximum likelihood method with 1,000 bootstrap replicates in MEGA 7.0.14 software. The yellow branches represent the NV GI sequences associated with the current diarrheal outbreak.

In the ORF1 phylogenetic tree, the 11 NV GI sequences from this diarrheal outbreak formed an evolutionary cluster positioned within the GI.P11 evolutionary branch, as shown in Figure 3. In the ORF2 phylogenetic tree, the 11 NV GI sequences from this diarrheal outbreak also formed an evolutionary cluster within the GI.6 evolutionary branch, depicted in Figure 4. In the ORF3 phylogenetic tree, the 11 NV GI sequences from this diarrheal outbreak formed an evolutionary cluster that is located on the same evolutionary branch as the NV GI.6 [P11] sequences, as illustrated in Figure 5.

#### 3.2.3 Gene variation analysis

The ORF1 of NV encodes a polyprotein that can be cleaved into p48, NTPase, p22, VPg, Pro, and RdRp, while ORF2 encodes VP1 and ORF3 encodes VP2, with VP1 and VP2 being structural proteins of NV. Compared to the isolated strain MW243609.1 from Shanxi, China, the p48 protein exhibited a Q137L mutation, where Q137 is located in the H-box/NC domain of p48. The H-box/NC domain is involved in regulating lipid synthesis and metabolism by influencing the formation of enlarged lipid droplets (Hung et al., 2023).

NTPase possesses RNA helicase activity, with motifs A (aa162**–**169), B (aa206**–**213), and C (aa245**–**259) representing the helicase core domains of NTPase (Li et al., 2018). In the 11 NV GI strains from this diarrheal outbreak, no mutations were found at the amino acid sites in the helicase core domains. NTPase contains two lipid droplet targeting motifs (LTM), with LTM-2 located at amino acids 51**–**90, which plays a role in NTPase's ER localization and the induction of cellular apoptosis (Yen et al., 2021). In the NV GI strains from this outbreak, a mutation E67D was observed in the LTM-2 of NTPase.

The amino acids at positions 65**–**72 of the p22 protein form the YXWESDG motif, which is essential for p22′s entry into COPII vesicles (Sharp et al., 2012, 2010). In this outbreak, the MERES motif of p22 did not exhibit any mutations. The amino acids at positions 112**–**127 of p22 comprise the membrane association domain (MAD), a critical domain for the localization of p22 to the Golgi apparatus, and this domain also remained unchanged. Notably, a mutation A162S was identified in p22, and analysis using NetPhos-3.1 indicated that S162 acts as a phosphorylation site, leading to the introduction of an additional phosphorylation site S162 in the p22 of the 11 NV GI strains from this outbreak.

During the NV replication process, the conserved tyrosine (Y26 in Murine Norovirus VPg and Y30 in Human noroviruses) in VPg binds to the 5′ terminus of the genomic RNAs and serves as a protein primer for the first base of RNA polymerase extension (Eden et al., 2011; Subba-Reddy et al., 2011; Leen et al., 2013). A stretch of 20 amino acids at the C terminus of NV VPg can initiate RNA translation by interacting with translation initiation factors (Leen et al., 2016; Daughenbaugh et al., 2003, 2006). In the 11 NV GI strains from this outbreak, neither Y30 nor the C terminus of VPg exhibited mutations. However, an S26N mutation occurred in VPg, with S26 identified as a phosphorylation site, resulting in the loss of the phosphorylation site S26 in the NV GI strains from this outbreak.

RdRp acts as the polymerase of NV, and the residues R182, D242, S300, N309, D343, and D344 are crucial for its enzymatic activity (Zamyatkin et al., 2009, 2008; Deval et al., 2017). No mutations were detected at these polymerase active sites in the 11 NV GI strains from this outbreak. However, a T133I mutation was found in the RdRp of one NV GI strain, changing residues 131–133 from NGT to NGI. Analysis using NetNGlyc-1.0 showed that the NV GI with the T133I mutation lost the N131 glycosylation site.

VP1 is the major structural protein of NV, with amino acids 337–346, 351–360, 366–377, 386–394, and 431–442 serving as binding sites for human monoclonal antibodies (Kimura-Someya et al., 2024). The histo-blood group antigens (HBGAs) function as receptors for NV, with receptor binding sites located at D331, H333, Q347, W384, S386, and S389 on NV GI (Tan and Jiang, 2010). In this diarrheal outbreak, NV GI exhibited S58N and R293G mutations; however, neither the binding sites for human monoclonal antibodies nor the receptor binding sites were affected (Table 2).

**Table 2:** Analysis of variant sites in 11 NV GI protein strains associated with the current diarrheal outbreak.

**Protein name**	**Variant sites of the 11 NV GI protein strains from the current diarrheal outbreak compared to the Chinese isolate MW243609.1 from Shanxi**
p48	Q137L (11)
NTPase	E67D (11)
p22	A162S (11)
VPg	S26N (11)
RdRp	T133I (1)
VP1	S58N (11), R293G (1)

The numbers in parentheses indicate the count of mutations occurring in the 11 NV strains associated with the current diarrheal outbreak.

### 3.3 SaV whole genome sequencing analysis

#### 3.3.1 Similarity analysis

Two strains of SaV sequences were obtained from the stool samples of patients involved in the diarrhea outbreak; one strain has a sequence length of 7,389 bp, while the other has a sequence length of 6,714 bp (lacking the *ORF2* gene sequence). Unfortunately, this study did not obtain the SaV genomic sequences from the water samples. Between the two SaV sequences from the diarrhea outbreak, there are two nucleotide site differences in the *ORF1* gene, with a similarity of 99.97%. The only differences in the encoded proteins of the two SaV sequences are found in NS1 and NS6–7, with similarities of 97.00% and 99.53%, respectively, while the amino acid sequences of NS2, NS3, NS4, NS5, and VP1 show 100% similarity, indicating they are completely identical.

In the BLAST analysis from the NCBI database, the two SaV sequences from this diarrhea outbreak are most similar to the Sapovirus GI strain Hu/GI.6/CHN/2021/RC-21020, which was isolated in Jiangsu Province, China, in February 2021. Compared to the Sapovirus GI strain Hu/GI.6/CHN/2021/RC-21020, the gene sequence of the outbreak Sapovirus GI strain Hu/GI.6/CHN/2024/JN01 has a whole genomic nucleotide sequence similarity of 98.38%, with an ORF2 gene nucleotide similarity of 97.99%. Additionally, the similarity of the SaV ORF1 between these two strains in the diarrhea outbreak is between 98.38% and 98.41% (Table 3).

**Table 3:** Similarity analysis of SaV genes.

**Gene**		**Similarity of SaV sequences between two strains in the current diarrheal outbreak**		**Similarity of SaV sequences from the current diarrheal outbreak with Sapovirus GI strain Hu/GI.6/CHN/2021/RC-21020**	
**Gene name**	**Protein name**	**Nucleotide**	**Amino acid**	**Nucleotide**	**Amino acid**
Complete Genome Sequence		-	-	98.38%	-
ORF1	NS1	99.97%	100%	98.38%−98.41%	97.00%
	NS2		100%		100%
	NS3		100%		100%
	NS4		100%		100%
	NS5		100%		100%
	NS6-7		100%		99.53%
	VP1		100%		100%
ORF2	VP2	-	-	97.99%	99.39%

“-” indicates that the sequence of one strain from this outbreak did not yield the ORF2 sequence.

#### 3.3.2 Genetic evolution analysis

**Figure 6:**
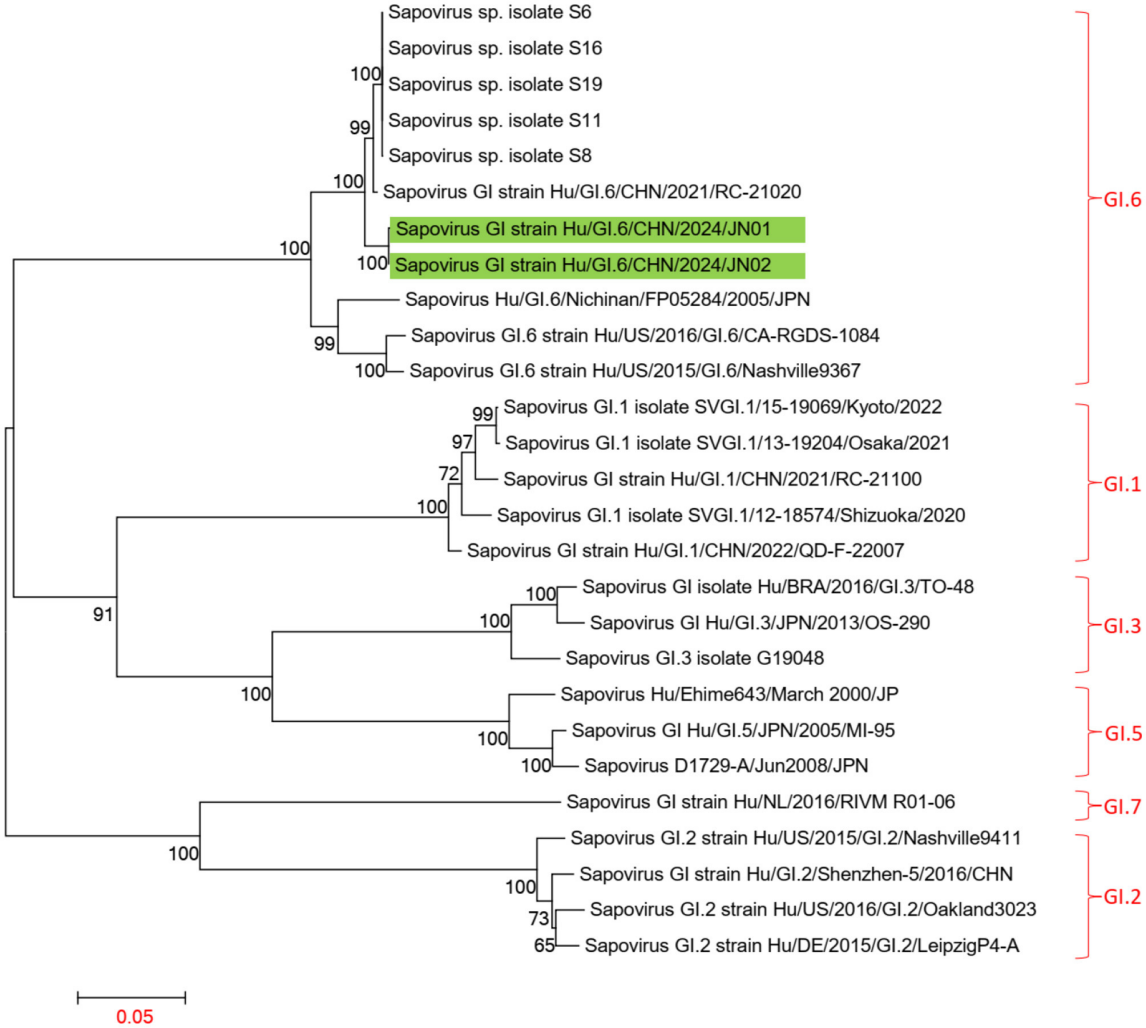
Phylogenetic tree of the complete genome of SaV GI. The tree was constructed using the maximum likelihood method with 1,000 bootstrap replicates in MEGA 7.0.14 software. The green branches represent the SaV sequences associated with the current diarrheal outbreak.

**Figure 7:**
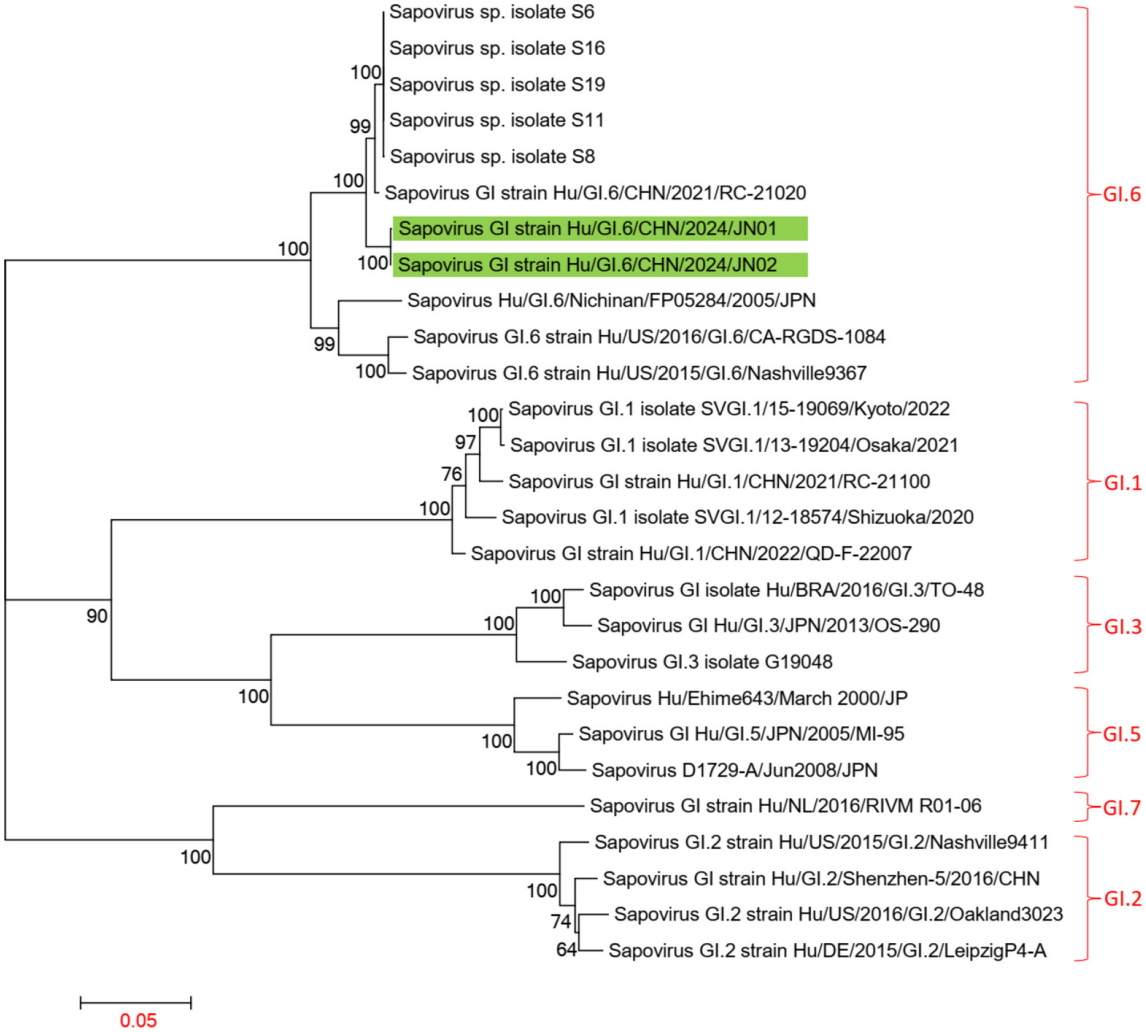
Phylogenetic tree of ORF1 for SaV GI. The tree was constructed using the maximum likelihood method with 1,000 bootstrap replicates in MEGA 7.0.14 software. The green branches indicate the SaV sequences associated with the current diarrheal outbreak.

**Figure 8:**
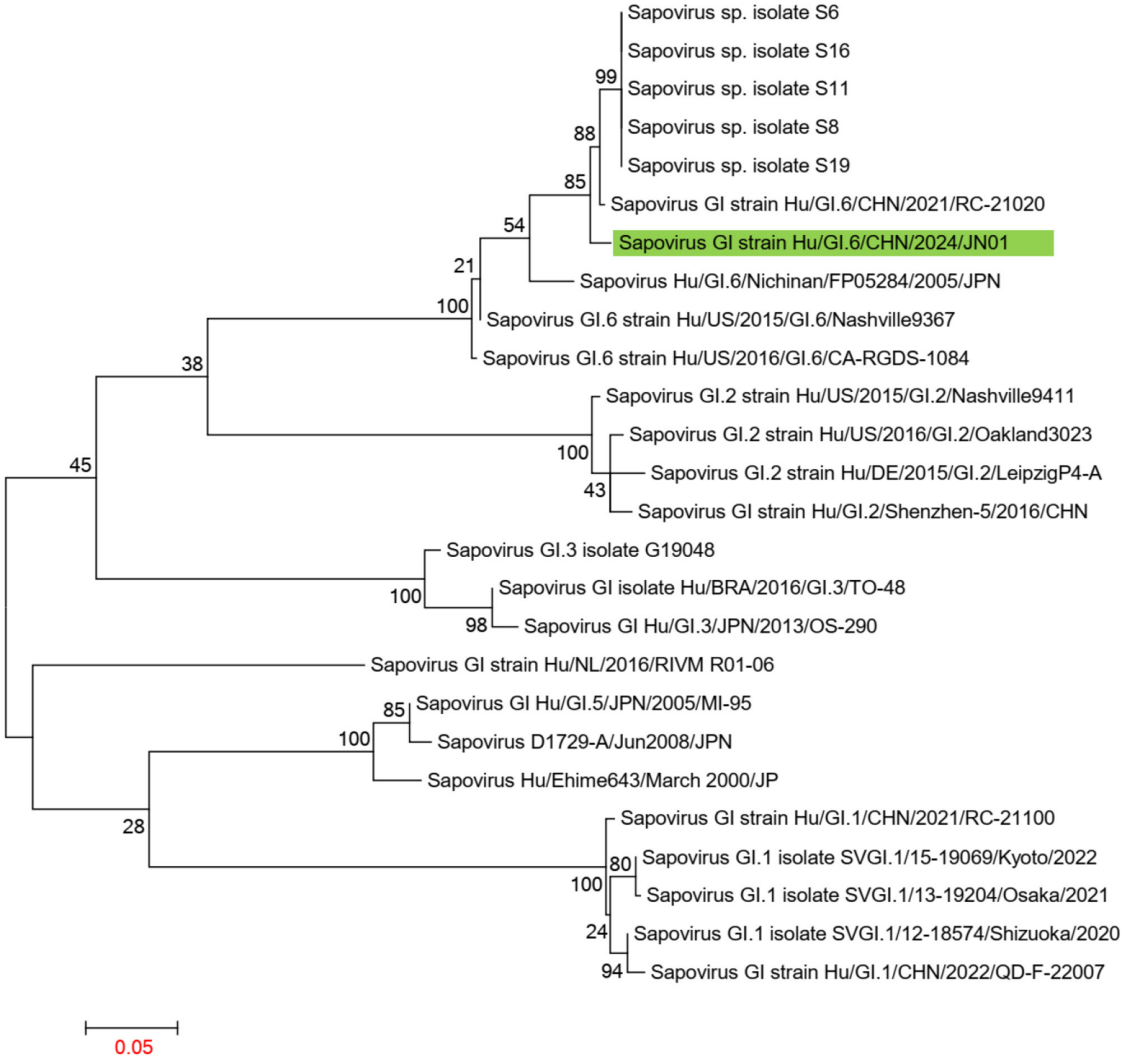
Phylogenetic tree of ORF2 for SaV GI. The tree was constructed using the maximum likelihood method with 1,000 bootstrap replicates in MEGA 7.0.14 software. The green branches represent the SaV sequences associated with the current diarrheal outbreak.

Using the Human Calicivirus Typing Tool, the genotypes of the two *SaV* gene sequences from this outbreak were analyzed, and both strains were found to belong to the GI.6 genotype. Phylogenetic trees for the complete SaV genome, ORF1, and ORF2 were constructed using the sequences of SaV GI.1, GI.2, GI.3, GI.5, GI.6, and GI.7 genotypes. The phylogenetic trees for the complete genome, ORF1, and ORF2 of SaV showed a high degree of similarity. In the phylogenetic trees of the complete SaV genome and ORF1, both strains from the diarrhea outbreak were located on the GI.6 evolutionary branch, and they independently formed an evolutionary cluster. This can be seen in Figures 6, 7. In the ORF2 phylogenetic tree, the sequences from this diarrhea outbreak were positioned on the same evolutionary branch as those of the SaV GI.6 genotype (Figure 8).

#### 3.3.3 Gene variation analysis

The SaV ORF1 encodes a polyprotein that can be cleaved into NS1, NS2, NS3, NS4, NS5, NS6-7, and VP1, while ORF2 encodes VP2. VP1 and VP2 are the structural proteins of SaV. NS6-7 contains the virus's chymotrypsin-like protease domain and RNA-dependent RNA polymerase domain. Among these, NS1 is known to be one of the least conserved proteins of SaV.

Compared to the Sapovirus GI strain Hu/GI.6/CHN/2021/RC-21020, the NS1 of the SaV involved in this diarrhea outbreak exhibited two amino acid mutations. Similarly, NS6-7 also showed two amino acid mutations. The H31, E52, and C116 in NS6-7 are the protease catalytic sites, while V32, T111, K113, G114, A132, G133, and T134 are part of the protease active site (Robel et al., 2008; Oka et al., 2007). However, no mutations were found at the protease catalytic sites and active sites in the two SaV strains from the diarrhea outbreak. The palm region and thumb domain of NS6-7 are associated with polymerase activity (Fullerton et al., 2007). The amino acid T554 is located in the extended loop domain of the polymerase, while E613 resides in the α-helix 14 domain of the polymerase, both of which are not included within the active domains of the enzyme. Phosphorylation analysis conducted using the NetPhos3.1 Server indicated that the two SaV strains from this diarrhea outbreak lost the phosphorylation site at T554. VP2 is known as the minor structural protein of the SaV virion, although its structure and function remain unclear (Li et al., 2022). In the SaV strain Hu/GI.6/CHN/2024/JN01 from this diarrhea outbreak, an A153T mutation was identified (Table 4).

**Table 4:** Analysis of variant sites in 2 SaV protein strains associated with the current diarrheal outbreak.

**Protein**	**Compared to Sapovirus GI Strain Hu/GI.6/CHN/2021/RC-21020, the variant sites of SaV associated with the current diarrheal outbreak show differences in comparison to Sapovirus GI Strain Hu/GI.6/CHN/2021/RC-21020**
NS1	L6F (2), T45A (2)
NS6-7	V42A (2), T554S (2), E613Q (2)
VP2	A153T

The numbers in parentheses indicate the number of mutations observed in the two SaV strains associated with the current diarrheal outbreak. VP2 represents the mutation sites in the diarrheal outbreak strain Sapovirus GI Strain Hu/GI.6/CHN/2024/JN01, while the VP2 sequence for the diarrheal outbreak strain Sapovirus GI Strain Hu/GI.6/CHN/2024/JN02 has not been obtained.

## 4 Discussion

NV and SaV are significant pathogens responsible for diarrheal outbreaks. In China, diarrheal outbreaks primarily occur in schools, with the GII.2 [P16] genotype being the main type associated with NV-related outbreaks, while the GI.6 [P11] genotype accounts for only 1.3% of NV-induced outbreaks (Jin et al., 2020). The genotypes of SaV causing diarrheal outbreaks differ across various regions of China: GI.6 is predominantly associated with outbreaks in Zhejiang province (Su et al., 2023), while Beijing mainly sees GI.1 and GI.2 genotypes (Jiao et al., 2023), and Shenzhen is primarily affected by GI.2 and GII.3 genotypes (Cheng et al., 2023).

This study conducted an epidemiological investigation, pathogen identification, and whole-genome sequencing analysis of a diarrheal outbreak that occurred after heavy rainfall. The results indicated that 26 individuals exhibited symptoms of diarrhea, with 21 stool specimens collected, among which 18 tested positive for either NV or SaV. Additionally, NV GI and SaV were detected in two samples of self-sourced well water. The outbreak may have resulted from pollution of the well water due to sewage backflow or leakage following the rainfall, leading to NV GI and SaV transmission between individuals. The outbreak suggests that there may be a certain level of distribution of rare genotypes, such as NV GI.6 [P11] and SaV GI.6, in the environment. Under specific conditions, such as heavy rainfall, enhanced environmental monitoring should be implemented to provide early warnings for NV and SaV outbreaks.

In Jining, China, GII genotypes of NV are primarily prevalent, with GI genotypes being relatively rare; however, the 11 complete genomic sequences of NV obtained in this outbreak were all identified as NV GI.6 [P11]. Phylogenetic trees constructed from the whole genome sequence, along with ORF1, ORF2, and ORF3 sequences, revealed that these 11 NV GI strains formed a distinct evolutionary cluster. Compared to the isolated strain MW243609.1 from Shanxi, the 11 NV GI strains from this outbreak exhibited no amino acid variations in Pro and VP2, while differences were observed in the amino acid sequences of P48, NTPase, p22, VPg, RdRp, and VP1. Notably, the polymerase active site of RdRp, the receptor binding sites of VP1 for human monoclonal antibodies, and the critical amino acids Y30 and C terminal of VPg involved in replication and translation remained unchanged. Conversely, amino acid mutations were detected in the H-box/NC domain of p48, which is involved in regulating lipid synthesis and metabolism, and in LTM-2 of NTPase related to ER localization and the induction of cellular apoptosis. Further research is needed to ascertain whether these mutations affect the functional characteristics of the associated proteins.

The two SaV strains obtained from this diarrheal outbreak were both identified as GI.6 genotype, displaying high homology. In phylogenetic trees of the whole genome and ORF1 sequences, these two SaV strains also formed a distinct evolutionary cluster. When compared to the Sapovirus GI strain Hu/GI.6/CHN/2021/RC-21020 from Jiangsu, none of the amino acid sequences of NS2, NS3, NS4, NS5, and VP1 underwent any changes, although some degree of amino acid mutations were observed in NS1, NS6-7, and VP2. Importantly, the protease catalytic sites in NS6-7 and the active structure domains of the polymerase remained unchanged.

Post-translational modifications (PTMs) can significantly impact viral replication and virulence. Phosphorylation and glycosylation are common PTMs in NV that affect processes such as replication and infection. However, research on the glycosylation and phosphorylation of SaV is relatively limited (Eden et al., 2011; Cheng et al., 2023; Mallagaray et al., 2019; Olspert et al., 2016). The NV strains from this diarrheal outbreak exhibited changes in glycosylation and phosphorylation patterns, including an increase in the phosphorylation site S162 in p22, loss of the S26 phosphorylation site in VPg, and loss of the N131 glycosylation site in RdRp. The NS6-7 of the two SaV strains lost the T554 phosphorylation site. The implications of these modifications on the functional characteristics of the related proteins remain unclear.

It is worth noting that although NV GI and SaV were detected in the self-sourced well water. The concentration of norovirus and SaV in the water samples from this outbreak may have been low. Only 500 mL of water was collected per sample, and large-volume samples were not taken, resulting in insufficient viral concentration after concentration, which hindered sequencing. The water samples only yielded a 75-bp gene sequence for NV, and this 75-bp fragment was identical to the sequences from 11 NV GI strains involved in the diarrhea outbreak. However, it showed some differences compared to the norovirus sequences in the NCBI database, providing supporting evidence that the norovirus in this outbreak may have been contaminated from the self-sourced well water. However, genomic sequences of SaV from the water samples were not obtained, preventing a comparative analysis between the genomic sequences of the outbreak cases and those from the well water. Rare genotypes such as NV GI.6 [P11] and SaV GI.6 are not the main types responsible for viral diarrhea. The immune antibody levels in the population against these genotypes may be low, and under specific conditions, these rare genotypes may cause an outbreak.

In conclusion, this study investigated a diarrheal outbreak that occurred in a school post-heavy rainfall, conducting epidemiological assessments, pathogen detection, and whole-genome sequencing analyses. The results confirmed that contamination of well water by NV GI and SaV, following the heavy rain, led to the outbreak. This further elucidates the correlation between heavy rainfall and the incidence of diarrhea. Therefore, enhancing awareness and preventive measures for diarrheal diseases during the rainy season should be prioritized to effectively prevent such outbreaks.

## Data Availability

The datasets presented in this study can be found in online repositories. The names of the repository/repositories and accession number(s) can be found in the article/supplementary material.
